# Laser-assisted selection of immotile spermatozoa has no effect on obstetric and neonatal outcomes of TESA-ICSI pregnancies

**DOI:** 10.1186/s12958-021-00835-9

**Published:** 2021-10-12

**Authors:** Huanhua Chen, Caizhu Wang, Hong Zhou, Jinhui Shu, Xianyou Gan, Kongrong Xu, Zhulian Wu, Xihe Deng, Guiting Huang, Ruoyun Lin

**Affiliations:** Reproductive Medicine Center, Maternal and Child Health Hospital of Guangxi Zhuang Autonomous Region, Nanning, 530003 China

**Keywords:** Laser, Immotile spermatozoa, Testicular sperm aspiration, Intracytoplasmic spermatozoa injection, Obstetric outcome

## Abstract

**Background:**

Azoospermic patients have benefited from both epididymal and testicular spermatozoa intracytoplasmic sperm injection (ICSI) treatment and lasers have been used to identify viable, immotile spermatozoa before the procedure. There are limited studies on the safety of laser-assisted selection of immotile spermatozoa. The aim of this study was to investigate the impact of laser-assisted selection of immotile spermatozoa on the obstetric and neonatal outcomes after ICSI.

**Methods:**

A retrospective comparative study was conducted on outcomes of ICSI cycles with testicular spermatozoa from June 2014 to June 2018. Of 132 cycles, 33 were allocated to the test group and oocytes were injected with immotile spermatozoa selected by laser, 99 cycles were allocated as control group.

**Results:**

Compared with the control group, no significant differences were found in the pregnancy, implantation, miscarriage and live birth rates in the test group in either fresh or frozen transfer cycles. The cumulative live birth rate in the test group was 69.70%, which was slightly higher than in the control group (60.61%), but this was not statistically different. There were no differences in the average gestational age, premature birth rate, neonatal birth weight, and the malformation rate between the test and control groups (*P* > 0.05). In addition, the obstetric outcome between the two groups were not different (*P* > 0.05).

**Conclusions:**

No negative effect on perinatal and neonatal outcomes was seen by using laser-assisted selection of immotile spermatozoa for TESA-ICSI. This study endorses the use of laser-assisted selection of viable spermatozoa for ICSI cycles.

## Background

Approximately 10 to 20% of infertile men suffer from azoospermia [1]. Such patients usually require surgery in order to obtain sperm, but the motility of spermatozoa are often quite low or even completely immotile [2, 3]. Embryologists often face a dilemma, not knowing how to choose spermatozoa for ICSI when encountering an absolute immotile sperm sample. Studies have shown that an oocyte injected with a live, immotile sperm can be successfully fertilized [4, 5], suggesting that sperm vitality is of greater importance to fertilization than motility.

Therefore, the question of how to select a live sperm from a number of immotile spermatozoa on the day of oocyte retrieval for ICSI is critical. Although the injection of completely immotile spermatozoa either from ejaculates or testicular biopsies can result in successful pregnancies and healthy babies [5, 6], there are many reports that the fertilization and embryo utilization rates are significantly lower when ICSI is performed with immotile compared to motile spermatozoa [7, 8]. Laboratory methods which can distinguish between viable but immotile and dead spermatozoa are necessary in order to provide convenient and cost effective treatment for patients.

Many approaches have been developed for detecting the viability of immotile sperm, and these include hypo-osmotic swelling (HOS) tests [9], use of chemicals for induction of tail movement [3] and laser [10, 11]. Studies have shown that the use of laser-assisted selection of viable but immotile spermatozoa for ICSI can provide better merits than conventional ICSI using other immotile sperm selection methods [12–14]. ICSI with viable but immotile spermatozoa selected by laser assessment could result in similar fertilization and embryo cleavage rates when compared with use of motile testicular spermatozoa [15]. Currently, very little data has been published on whether lasers can affect perinatal and neonatal outcomes of ICSI patients. The objective of this retrospective study was to evaluate whether the use of laser-assisted selection of viable but immotile testicular spermatozoa for ICSI affects the resultant pregnancy outcome.

## Patients and methods

### Patients

We performed a retrospective analysis of the outcomes of patients whose oocytes were fertilized by ICSI with sperm obtained by testicular aspiration (TESA) in our clinic from June 2014 to June 2018. The inclusion criteria included all TESA-ICSI cycles that used fresh or frozen-thawed testicular spermatozoa for oocytes injection. The exclusion criteria were as follows: cycles were cancelled for no embryos available, and the embryo transfer was performed on either days 1 or 2.

All included cases were divided into test and control groups according to whether motile spermatozoa were found during ICSI. In the test group, a laser was applied to select sperm for ICSI because there was no motile sperm. In the control group, routine ICSI was performed, and sperm were selected mainly based on motility and morphology.

### Ovarian stimulation

Ovarian stimulation was performed using a routine protocol developed by our clinic [16]. Briefly, all female patients were down-regulated by use of leuprolide acetate (Lupron; TAP Pharmaceuticals, Lake Forest, Illinois). Ovarian stimulation was achieved with the use of recombinant follicle stimulating hormone (FSH) (Gonal-F or Puregon; Merck Serono, Italy). When two or more follicles reached 18 mm in mean diameter, 5000–10,000 IU human chorionic gonadotropin (hCG) (Serono, Switzerland; or Livzon, China) was administered. Oocytes were collected by follicular aspiration with the use of vaginal ultrasonography 36 h after hCG administration.

### Testicular sperm aspiration

Testicular sperm aspiration was carried out mainly for those who were diagnosed with obstructive azoospermia and non-obstructive azoospermia. Where there was a failure to obtain sperm by masturbation such as cryptozoospermia, congenital absence of vas deferens, retrograde ejaculation or unexpected ejaculation failure on the day of oocyte retrieval, the male patients were also subjected to surgical aspiration.

The male patients were placed in the supine position and disinfected following routine procedures [14]. Anesthesia was performed by using 2% lidocaine to block the spermatic cord. A 50-mL syringe containing 0.5 mL of fertilization Quinn’s 1020 medium (Sage, Trumbull, CT, USA) and a 16-gauge needle were used for aspiration of the seminiferous tubules. The tubules were independently minced using two sterile needles in a culture dish containing 2 mL of Quinn’s 1020 medium. The processed samples were then observed under high magnification (× 200 magnification). If no motile sperm were found immediately or after 2 ~ 4 h of culture in a 6% carbon dioxide incubator maintained at 37 °C, the sperm were considered to be immotile.

### Laser selection of immotile spermatozoa

Using a protocol based on the method of Aktan et al [10], the tips of immotile sperm were targeted with a laser beam of approximately 200 μJ with an irradiation time of about 2 ms (RI Saturn 5™ Laser System, UK). Those spermatozoa which presented with curling of the tails after the laser shot were regarded as viable, while others which did not respond in this way were considered to be non-viable. The first criteria for sperm selection was viable sperm. If the number of viable sperm was sufficient for oocyte insemination, further selection was made based on sperm morphology.

### ICSI

All ICSI procedures were performed 39–40 h after hCG administration. After ICSI, oocytes were transferred to culture dishes and kept in fertilization medium (Quinn Advantage medium, ART-1020) supplemented with 10% Quinn Advantage serum protein substitute (SPS, ART-3010; Sage) until the time of pronuclear observation.

### Embryo culture and embryo transfer

Fertilization was confirmed at 16–18 h after ICSI. Only zygotes displaying two pronuclear bodies were counted and transferred to the cleavage culture media (Quinn Advantage medium, ART-1026) supplemented with 10% SPS for further culture. The day of ICSI manipulation was considered as day 0. On day 3, the cleavage embryos were scored. Top scoring embryos on day 3 were defined as cell number ≥ 6, fragmentation rate of less than 20%, evenly sized blastomeres and no vacuolization. The patients with good prognosis who **(**defined as maternal age ≤ 35 years, receiving first or second ART cycle and with more than three top embryos on day 3) were advised to delay culture until days 5 or 6, and a single blastocyst was chosen for transfer. Those patients with a poor prognosis underwent day 3 embryo transfers. If a fresh day 3 embryo transfer was cancelled, the top scoring embryos were vitrified. Then the remaining embryos after transfer or vitrification from patients with a poor prognosis were also delayed in culture until days 5 or 6. Blastocyst culture media (Quinn Advantage medium, ART-1029) supplemented with 10% SPS was used for this. The supernumerary usable blastocysts from both patients with good and poor prognosis were vitrified.

The embryo transfer strategy used in our clinic was as follows: (i) for the good prognosis patients, a single blastocyst was transferred in either fresh or frozen-thawed cycles. (ii) for the patients with a poor prognosis, one D3 embryo was transferred if the woman was diagnosed with a scarred uterus or the patient’s height was less than 150 cm, while others underwent double D3 embryo transfers.

### Preparation of the endometrium for frozen-thawed transfer cycle

The patients who underwent frozen-thawed embryo transfers were: (i) those who had cancelled fresh embryo transfer (ii) those who failed to conceive during the fresh cycle or (iii) those who desired to conceive a subsequent child.

The endometrium preparation protocol used was according to the methods established in our clinic [17]. Natural or hormone replacement cycles were commonly used for endometrial preparation. During natural cycles, a progesterone (P) intramuscular injection (60 mg per day) was administrated when follicular ovulation or the luteinizing hormone (LH) peak were detected. Ultrasound-guided embryo transfers were performed using a catheter on day 5 for blastocysts or on day 3 for cleavage embryos, as timed after ovulation. In hormone replacement cycles, the patients began to take oral oestradiol valerate (4-6 mg per day) on menstrual cycle day 3. P intramuscular injection (60-100 mg) was administrated when the endometrial thickness reached 7-8 mm, oestradiol (E_2_) ≥ 400 pmol/L and *P* ≤ 4.77 nmol/L. Embryo transfers were performed on day 6 for blastocysts or on day 4 for cleavage embryos after progesterone intramuscular injection. After transfer, vaginal progesterone at a dose of 0.2 g was administered three times per day as a routine scheme for luteal support.

### Blastocyst or cleavage embryo cryopreservation and thawing procedures

The procedures of blastocyst vitrification and thawing were carried out according to the methods established by our team group and described previously [17]. The media used for freezing and thawing of blastocysts were prepared in house. Before freezing, the blastocysts were artificially shrunk, then moved into the HEPES-buffered culture medium (Quinn’s-1023, SAGE, USA) supplemented with 20% human serum albumin (HSA, SAGE, USA) and washed for 30 s. Subsequently, they were placed into the equilibration solution containing 10% (v/v) ethylene glycol (American Sigma) and 10% (v/v) DMSO (American Sigma) for 1 min in order to equilibrate. After that, the blastocysts were exposed to a cryoprotectant solution containing 20% (v/v) ethylene glycol, 20% (v/v) DMSO and 0.3 mol/L sucrose (American Sigma) and incubated for a further 30 s. Each Cryotop (Kitazato, Japan) containing one blastocyst was plunged immediately into liquid nitrogen.

For warming, the thawing solutions (TS) used were kept at 37 °C. The blastocysts were rapidly warmed in the TS1 containing 0.6 mol/L sucrose in HEPES-buffered media supplemented with 20% HSA and incubated for 2 min, followed by TS2 containing 0.5 mol/L sucrose for 3 min and then TS3 containing 0.25 mol/L sucrose for a further 3 min. Finally, the blastocysts were washed in the HEPES-buffered media with 20% HSA. Blastocysts were thawed 1-3 h before transfer and allowed to incubate equilibrate for re-expansion purposes.

For cleavage embryo cryopreservation, only top scoring embryos on day 3 were considered for vitrification. The vitrification kit and the devices used for the frozen-thawed cycles were all purchased from Kitazato (Japan, http://www.kitazato.co.jp/). Vitrification was performed at room temperature. The embryos were placed into the equilibration solution (ES) and allowed to equilibrate 8-10 min. After that, the embryos were washed in vitrification solution (VS) for 1 min, and then they were placed on the film strip of the Cryotop, and plunged into liquid nitrogen immediately for cryopreservation.

On the day of transfer in frozen-thawed cycles, embryos were warmed. A set of tubes purchased from Kitazato, Japan, containing four different thawing solutions (TS, DS, WS1 and WS2) was used. Before warming, TS was incubated in a 37 °C incubator for 30 min, and DS, WS1, and WS2 were left at room temperature. The Cryotop devices containing the embryos were removed and directly immersed in TS for less than 1 min. The embryos were immediately transferred to DS for 3 min, followed by incubation in WS1 and in WS2 for 5 min each, respectively. After thawing, the embryos were transferred into the blastocyst culture medium and cultured for 2-4 h before transfer.

### Follow-up and evaluation indices

Serum levels of βhCG were measured on the 14th day post-transfer. Clinical pregnancies were confirmed by transvaginal ultrasonography imaging of the presence of gestational sac within the uterine cavity 28 days after transfer. The primary outcome was cumulative live birth rate which was calculated by per oocyte retrieval cycle. It was calculated by the number of live births over a period for each oocyte retrieval cycle (including live birth from both fresh and frozen embryo transfer cycles, and when the number of live births was greater than or equal to 2 in one oocyte retrieval cycle was considered to be one) divided by the total number of oocyte retrieval cycles. The data collecting for cumulative live birth rate calculating were included up to December 2019. Birth weight lower than 2500 g was defined as low birth weight. Preterm birth was defined as < 37 weeks gestational age.

### Statistical analysis

Statistical analysis was performed with the use of the Student t test and chi-squared analysis. *P* < 0.05 was considered to be statistically significant.

## Results

**Fig. 1:**
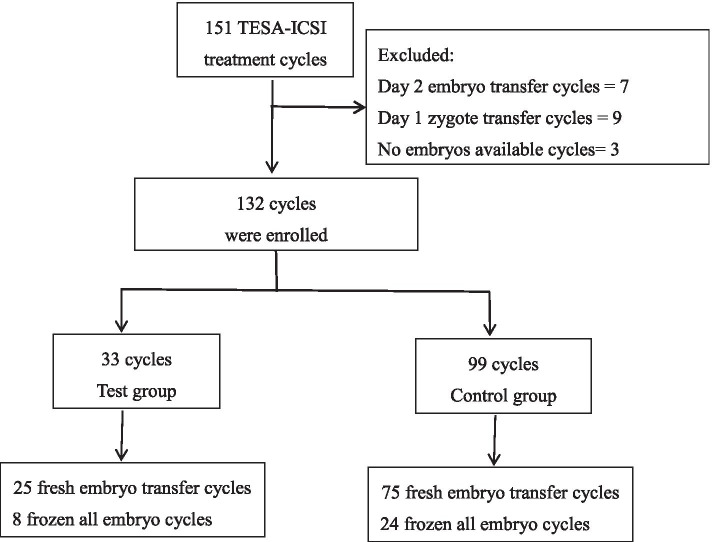
Flow chart of ICSI treatment cycles included in this study

The final data analysis included 132 ICSI cycles of which there were 33 cycles in the test group and 99 cycles in the control groups (Fig. 1). The main characteristics of the female patients and the male infertility factors relating to the reasons for undergoing testicular sperm aspiration are shown in Tables 1 and 2, respectively. As shown in Table 1, the mean age, average cycles attempts, BMI, infertility duration, the levels of baseline FSH and LH in fresh cycles were not statistically different between the test and control groups (*P* > 0.05). Table 2 shows that there were no differences in male infertility factors that led to the use of testicular sperm aspiration between the two groups (*P* > 0.05).

**Table 1:** Characteristics of the female patients in this study

	Test group	Control group	*P* value
Oocyte retrieval cycles	33	99	
Average cycle attempts	1.21 ± 0.65	1.18 ± 0.52	0.787
Age (years)	30.88 ± 5.52	30.97 ± 5.53	0.935
Infertility duration (years)	4.70 ± 2.90	4.64 ± 3.81	0.565
Maternal body mass index (kg/m^2^)	20.96 ± 2.40	21.66 ± 3.31	0.265
Baseline FSH (IU/L)	6.69 ± 1.38	7.50 ± 2.95	0.132
Baseline LH (IU/L)	5.35 ± 2.94	5.45 ± 2.60	0.855

**Table 2:** Male infertility factors that led to the use of testicular sperm aspiration

	Test group	Control group	*P* value
Oocyte retrieval cycles	33	99	
Age (years)	33.91 ± 6.87	33.64 ± 5.82	0.824
Obstructive azoospermia n (%)	21 (63.64%)	66 (66.67%)	0.833
Non-obstructive azoospermia n (%)	1 (3.03%)	9 (9.09%)	0.450
Severe oligospermia n (%)	3 (9.09%)	3 (3.03%)	0.165
Cryptozoospermia n (%)	2 (6.06%)	0	0.061
Unexpected ejaculation failure n (%)	3 (9.09%)	5 (5.05%)	0.412
Anejaculation n (%)	3 (9.09%)	8 (8.08%)	1.000
Congenital absence of vas deferens n (%)	0	2 (2.02%)	1.000
Spermatogenic dysfunction n (%)	0	1 (1.01%)	1.000
Retrograde ejaculation n (%)	0	4 (4.04%)	0.572
Y chromosome microdeletion (SY127 in AZFb region) n (%)	0	1 (1.01%)	1.000

No differences were found in terms of fertilization rate (78.17% vs 80.48%), cleavage rate (95.76% vs 96.59%), top embryo rate on day 3 (44.65% vs 43.14%), and embryo utilization rate (46.49% vs 50.07%) between the test group and control group (*P* > 0.05). In the fresh embryo transfer cycles, the clinical pregnancy rate (64.00% vs 45.33%), implantation rate (45.95% vs 34.86%), miscarriage rate (12.50% vs 11.76%) and live birth rate (56.00% vs 38.67%) were not statistically different between the test and control groups (*P* > 0.05) (Table 3).

**Table 3:** Comparison of embryo culture and pregnancy outcomes between the test and control groups

	Test group	Control group	*P* value
Oocyte retrieval cycles	33	99	
Mean no. of oocytes retrieved	14.36 ± 7.80	12.82 ± 6.67	0.272
Fertilization rate	78.17% (283/362)	80.48% (763/948)	0.352
Cleavage rate	95.76% (271/283)	96.59% (737/763)	0.523
Top embryos rate on day 3	44.65% (121/271)	43.14% (318/737)	0.670
Embryo utilization rate	46.49% (126/271)	50.07% (369/737)	0.321
Fresh embryo transfer cycles, n	25	75	
Endometrial thickness (mm)	11.74 ± 2.74	11.62 ± 2.20	0.827
Average number of embryos transferred	1.48 ± 0.59	1.45 ± 0.53	0.832
Proportion of blastocyst transfer cycles	44.00%(11/25)	37.33%(28/75)	0.638
Clinical pregnancy rate	64.00% (16/25)	45.33% (34/75)	0.165
Implantation rate	45.95% (17/37)	34.86% (38/109)	0.244
Miscarriage rate	12.50% (2/16)	11.76% (4/34)	1.000
Live birth rate	56.00% (14/25)	38.67% (29/75)	0.163
Frozen embryo transfer cycles, n	24	85	
Endometrial thickness (mm)	9.85 ± 1.75	9.33 ± 1.77	0.205
Proportion of blastocyst transfer cycles	83.33%(20/24)	80.00%(68/85)	1.000
Clinical pregnancy rate	58.33% (14/24)	49.41% (42/85)	0.494
Implantation rate	53.57% (15/28)	46.24% (43/93)	0.524
Live birth rate	41.67% (10/24)	40.00% (34/85)	1.000
Cumulative live birth rate	69.70% (23/33)	60.61% (60/99)	0.409

In the frozen-thawed transfer cycles, there were no statistically significant differences in the clinical pregnancy rate (58.33% vs 49.41%), implantation rate (53.57% vs 46.24%) and live birth rate (41.67% vs 40.00%) between the test and control groups (Table 3).

The cumulative live birth rate of the test group was higher than that of the control group (69.70% vs 60.61%), but was also not significantly different (*P* > 0.05) (Table 3).

Table 4 shows the neonatal outcomes. A total of 94 babies including of 6 twin babies in the test group and 8 twin babies in the control group were born. No significant differences with respect to method of delivery were seen. In addition, no differences were observed in the mean gestational age (38.26 weeks ±1.28 vs 38.37 weeks ±1.35), preterm delivery rate (11.11% vs 7.46%), mean birth weight at delivery (2894.82 g ± 623.32 vs 3101.34 g ± 435.04) and malformation rate (0.00% vs 1.49%) between the test and control groups, respectively (*P* > 0.05 in all cases). With respect to obstetric outcomes between the test and control groups, no significant differences were seen (*P* > 0.05) (Table 5).

**Table 4:** Comparison of neonatal outcomes between the test and control groups

	Test group	Control group	*P* value
Cumulative live birth babies	27	67	
Gestational weeks at delivery	38.26 ± 1.28	38.37 ± 1.35	0.987
Preterm delivery (< 37 weeks)	11.11% (3/27)	7.46% (5/67)	0.685
Birth weight (grams)	2894.82 ± 623.32	3101.34 ± 435.04	0.071
Birth weight < 2500 g	14.80% (4/27)	8.96% (6/67)	0.465
Birth weight > 4000 g	3.70% (1/27)	1.49% (1/67)	0.494
Malformation rate	0	1.49% (1/67)	1.000

**Table 5:** Comparison of obstetric outcomes between the test and control groups

	Test group	Control group	*P* value
Total deliveries cycles	24	63	
Cesarean delivery	45.83% (11/24)	52.38% (33/63)	0.637
Gestational hypertension	8.33% (2/24)	3.17% (2/63)	0.304
Gestational Diabetes	12.5% (3/24)	6.35% (4/63)	0.389
Premature rupture of membranes	8.33% (2/24)	6.35% (4/63)	0.666
Placenta previa	0.00% (0/24)	1.59% (1/63)	1.000
Postpartum hemorrhage	0.00% (0/24)	3.17% (2/63)	1.000
Fetal distress	16.67% (4/24)	6.35% (4/63)	0.208
Premature birth	12.50% (3/24)	9.52% (6/63)	0.702
Low birth weight	16.67% (4/24)	12.70% (8/63)	0.730

## Discussion

Our study was undertaken to investigate the effectiveness and safety of lasers for the identification of viable but immotile spermatozoa in TESA-ICSI cycles. We found that there were no statistical differences in embryo development and pregnancy outcomes between the test and control groups. We also confirmed that there were no negative effects on obstetric and neonatal outcomes by using laser assisted selection of viable but immotile spermatozoa. This is the first study to focus on the obstetric and neonatal outcomes after ICSI using immotile spermatozoa selected by laser technology.

Azoospermic males have previously benefited from the retrieval of spermatozoa by using testicular sperm aspiration, testicular sperm extraction, or and micro-testicular sperm extraction surgery. The use of these procedures can result in a few motile sperm and in some cases only immotile spermatozoa are retrieved [8]. The selection of a viable spermatozoon for ICSI is an essential prerequisite in order to achieve fertilization and optimal pregnancy rates. The embryologist often struggles to find enough viable spermatozoa for the ICSI protocol. Several methods have been developed to distinguish viable spermatozoa from the immotile fraction, but each have their own set of advantages and disadvantages [12]. In addition, any chemicals used during the selection process may have adverse effects on the development of the embryo as well as the outcome of the pregnancy. Thus, a quick, easy and safe technique for selection of a suitable candidate immotile spermatozoon for ICSI would be welcomed by both the clinician and the patient.

In recent years, laser has been widely used in the field of assisted reproductive technology, including assisted hatching [18, 19], embryo biopsies [20] and sperm immobilization [21]. The clinical use of sperm selection with a laser has recently gained more attention. The ability of laser technology to identify viable spermatozoa was first reported by Aktan et al. [10]. Using this technique, the oocytes were achieved higher fertilization and cleavage rates in cases with fresh testicular spermatozoa as well as in cases with ejaculated sperm. Successful pregnancies were obtained by several groups using a laser to select of viable spermatozoa before ICSI [22, 23]. In addition, it was reported that the use of the laser in selecting immotile testicular spermatozoa and the implementation of the zona score and spindle visualization significantly increased the fertilization rate of the testicular sperm extraction (TESE) ICSI program [24].

Previous studies were mainly focused on the fertilization rate, whereas systematic research on clinical and neonatal outcomes after laser-assisted selection of immotile sperm is not well documented. In our study, it was gratifying to find that there were no significant differences in the fertilization and cleavage rates as well as the rate of high quality day 3 embryos between the test and control groups. Furthermore, the clinical pregnancy, implantation and live birth rates were not significantly different in both the fresh and frozen-thawed transfer cycles between the two groups. The cumulative live birth rate of test group was slightly higher than that of control group although this did not reach statistical significance.

The safety of assisted reproductive technology treatment is always a concern. A number of studies have reported that in vitro fertilization (IVF) or ICSI -conceived offspring, even if they are singleton pregnancies, are associated with low birth weights and preterm deliveries [25, 26]. Meta-analysis studies have also concluded that children conceived from IVF and ICSI can present with an increased risk for congenital malformations compared with those naturally conceived, although these risks did not differ between IVF and ICSI [27–29]. Concerns with respect to the safety of using immotile spermatozoa for ICSI have arisen mainly as some assisted methods have used chemical substances to select viable spermatozoa [30, 31]. The exact biochemical effects of these compounds on human spermatozoa and embryos are not well demonstrated.

Studies have confirmed that laser-assisted operation in embryo hatching, embryo biopsies and sperm immobilization did not appear to increase the risk of adverse neonatal outcomes [32–34]. The use of a laser to determine spermatozoa viability is generally based on sperm protein activities and the integrity of the tail membranes. In theory, a single laser shot applied to the far end of the flagellum of a viable spermatozoon should not cause any adverse effects on the genetic material [35]. Moreover, using a laser beam to select spermatozoa does not require the use of any chemical substances to either induce spermatozoa motility or cause spermatozoa flagellum curling. Laser-assisted selection of viable but immotile spermatozoa can be directly used for ICSI in a petri dish, and the sperm can be injected immediately into the oocyte. Consequently, no accompanying side-effects are expected. In our study, no statistically significant differences in adverse obstetric and neonatal outcomes were found when the test group was compared to the control group. These results will provide encouraging evidence for embryologists to select viable but immotile spermatozoa by using laser technology.

There are some limitations in this study. Firstly, the sample size was too small to divide subgroups for independent analysis of the use of fresh and frozen-thawed spermatozoa. Although a systematic review and meta-analysis reported that no statistical differences were found in the fertilization and good quality embryo rates between the frozen-thawed immotile spermatozoa group and the routine fresh immotile spermatozoa ICSI group [36]. Larger samples are needed to compare the clinical outcomes of these two subgroups. Secondly, we did not perform sub-analysis to compare differences between cleavage embryo transfer and blastocyst transfer in clinical outcomes due to limitations of sample size. Some studies have shown that blastocyst transfer could be a preferred strategy to increase implantation, pregnancy and live birth rates, and is not associated with increased unfavorable obstetric and perinatal outcomes compared with cleavage-stage embryo transfer [37, 38]. Other studies reported no superiority of blastocyst compared with cleavage-stage embryo transfer in clinical practice [39, 40]. In our study, the proportion of patients who performed blastocysts transfer was similar between the two groups regardless of whether fresh or frozen cycles were used. Furthermore, there was no statistical difference in the average number of embryos transferred between the two groups. These results indicated that the embryo transfer strategy might have no effect on our conclusions. Thirdly, only neonatal outcome was collected and analyzed. The study would benefit from long term follow-up of children beyond neonatal stage. These shortcomings may be addressed in our future studies.

In conclusion, there was no statistical increase in the risk of obstetric and neonatal outcomes in the TESA ICSI cycles following laser-assisted selection of viable but immotile spermatozoa. Patients presented with viable but immotile spermatozoa will benefit from laser application in the ICSI program.

## Data Availability

Please contact author for data requests.

## Abbreviations

ART: Assisted reproductive technique
TESA: Testicular sperm aspiration
ICSI: Intracytoplasmic sperm injection
HOS: Hypo-osmotic swelling
hCG: Human chorionic gonadotropin
SPS: Serum protein substitute
TESE: Testicular sperm extraction
IVF: In vitro fertilization
BMI: Body mass index
FSH: Follicle stimulating hormone
LH: Luteinizing hormone
P: Progesterone
E_2_: Oestradiol.

## References

[1] Jarow JP, Espeland MA, Lipshultz LI (1989). Evaluation of the azoospermic patient. J Urol.

[2] Kovacic B, Vlaisavljevic V, Reljic M (2006). Clinical use of pentoxifylline for activation of immotile testicular sperm before ICSI in patients with azoospermia. J Androl.

[3] Gu YF, Zhou QW, Zhang SP, Lu CF, Gong F (2018). The clinical and neonatal outcomes after stimulation of immotile spermatozoa using SperMagic medium. Adrologia.

[4] Terriou P, Hans E, Giorgetti C, Spach JL, Salzmann J, Urrutia V (2000). Pentoxifylline initiates motility in spontaneously immotile epididymal and testicular spermatozoa and allows normal fertilization, pregnancy, and birth after intracytoplasmic sperm injection. J Assist Reprod Genet.

[5] Kaushal M, Baxi A (2007). Birth after intracytoplasmic sperm injection with use of testicular sperm from men with Kartagener or immotile cilia syndrome. Fertil Steril.

[6] Mclachlan RI, Ishikawa T, Osianlis T, Robinson P, Merriner DJ (2012). Normal live birth after testicular sperm extraction and intracytoplasmic sperm injection in variant primary ciliary dyskinesia with completely immotile sperm and structurally abnormal sperm tails. Fertil Steril.

[7] Esfandiari N, Javed MH, Gotlieb L, Casper RF (2005). Complete failed fertilization after intracytoplasmic sperm injection--analysis of 10 years’ data. Int J Fertil Womens Med.

[8] Stalf T, Mehnert C, Hajimohammad A, Manolopoulos K, Shen Y, Schuppe HC (2010). Influence of motility and vitality in intracytoplasmic sperm injection with ejaculated and testicular sperm. Andrologia..

[9] Zubair M, Ahmad M, Jamil H (2015). Review on the screening of semen by hypo-osmotic swelling test. Andrologia..

[10] Aktan TM, Montag M, Duman S, Gorkemli H, Yurdakul T (2004). Use of a laser to detect viable but immotile spermatozoa. Andrologia..

[11] Yazdi RS, Bakhshi S, Jannat Alipoor F, Akhoond MR, Borhani S, Farrahi F (2014). Effect of 830-nm diode laser irradiation on human sperm motility. Laser Med Sci..

[12] Nordhoff V (2015). How to select immotile but viable spermatozoa on the day of intracytoplasmic sperm injection? An embryologist's view. Andrology..

[13] Simopoulou M, Gkoles L, Bakas P, Giannelou P, Koutsilieris M (2016). Improving ICSI: a review from the spermatozoon perspective. Syst Biol Reprod Med.

[14] Chen HH, Zhou H, Shu JH, Gan XY, Wang CZ, Lin RY (2019). A point of confusion for embryologists in the identification of viable spermatozoa by the eosin-nigrosin test. Clin Exp Reprod Med..

[15] Chen HH, Feng GX, Zhang B, Zhou H, Wang CZ, Shu JH (2017). A new insight into male fertility preservation for patients with completely immotile spermatozoa. Reprod Biol Endocrinol.

[16] Wang CZ, Feng GX, Shu JH, Zhou H, Zhang B, Chen HH (2018). Cumulus oophorus complexes favor physiologic selection of spermatozoa for intracytoplasmic sperm injection. Fertil Steril.

[17] Wang CZ, Shu JH, Lin RY, Chen HH, Gan XY, Deng XH (2020). Choosing the optimal blastocyst by morphology score versus developmental rate in frozen-thawed embryo transfer cycles. Hum Fertil.

[18] Lu XM, Liu YB, Cao X, Liu SY, Dong X (2019). Laser-assisted hatching and clinical outcomes in frozen-thawed cleavage-embryo transfers of patients with previous repeated failure. Laser Med Sci..

[19] Ng C, Wais M, Nichols T, Garrow S, Hreinsson J, Luo ZC (2020). Assisted hatching of vitrified-warmed blastocysts prior to embryo transfer does not improve pregnancy outcomes. J Ovarian Res.

[20] Patrizia RMS, Lucia TBS, Alonso RRDA, Kohar MMS, Lisa GBS, Lindsay DBS (2020). Trophectoderm biopsy protocols can affect clinical outcomes: time to focus on the blastocyst biopsy technique. Fertil Steril.

[21] Ebner T, Yaman C, Moser M, Hartl J, Tews G (2001). Laser assisted immobilization of spermatozoa prior to intracytoplasmic sperm injection in humans. Hum Reprod.

[22] Gerber PA, Kruse R, Hirchenhain J, Krüssel J, Neumann NJ (2008). Pregnancy after laser-assisted selection of viable spermatozoa before intracytoplasmatic sperm injection in a couple with male primary cilia dyskinesia. Fertil Steril.

[23] Chen HH, Feng GX, Zhang B, Zhou H, Gan XY (2017). A successful pregnancy using completely immotile but viable frozen-thawed spermatozoa selected by laser. Clin Exp Reprod Med.

[24] Nordhoff V, Schüring AN, Krallmann C, Zitzmann M, Schlatt S, Kiesel L, Kliesch S (2013). Optimizing TESE-ICSI by laser-assisted selection of immotile spermatozoa and polarization microscopy for selection of oocytes. Andrology..

[25] Zhao J, Xu B, Zhang Q, Li YP (2016). Which one has a better obstetric and perinatal outcome in singleton pregnancy, IVF/ICSI or FET?: a systematic review and meta-analysis. Reprod Biol Endocrinol.

[26] Cavoretto PI, Giorgione V, Sotiriadis A, Vigano P, Papaleo E, Galdini A (2020). IVF/ICSI treatment and the risk of iatrogenic preterm birth in singleton pregnancies: systematic review and meta-analysis of cohort studies. J Matern Fetal Neonatal Med.

[27] Lacamara C, Ortega C, Villa S, Pommer R, Schwarze JE (2017). Are children born from singleton pregnancies conceived by ICSI at increased risk for congenital malformations when compared to children conceived naturally? A systematic review and meta-analysis. JBRA Assist Reprod.

[28] Zheng Z, Chen L, Yang T, Yu H, Wang H, Qin JB (2018). Multiple pregnancies achieved with IVF/ICSI and risk of specific congenital malformations: a meta-analysis of cohort studies. Reprod BioMed Online.

[29] Jin L, Li Z, Gu LJ, Huang B (2020). Neonatal outcome of children born after ICSI with epididymal or testicular sperm: a 10-year study in China. Sci Rep.

[30] Navas P, Paffoni A, Intra G, González-Utor A, Clavero A, Gonzalvo MC (2017). Obstetric and neo-natal outcomes of ICSI cycles using pentoxifylline to identify viable spermatozoa in patients with immotile spermatozoa. Reprod BioMed Online.

[31] Ebner T, Tews G, Mayer RB, Ziehr S, Arzt W, Costamoling W, Shebl O (2011). Pharmacological stimulation of sperm motility in frozen and thawed testicular sperm using the dimethylxanthine theophylline. Fertil Steril.

[32] Vizziello G, Carone D, Caroppo E, Vitti A, D'Amato G (2005). Laser assisted intracytoplasmic sperm injection: a more effective and faster technique of immobilization of spermatozoa than traditional one. Minerva Ginecol.

[33] He H, Jing S, Fu CF, Tan YQ, Luo KL, Zhang SP (2019). Neonatal outcomes of live births after blastocyst biopsy in preimplantation genetic testing cycles: a follow-up of 1,721 children. Fertil Steril.

[34] Zeng MF, Su SQ, Li LM (2018). The effect of laser-assisted hatching on pregnancy outcomes of cryopreserved-thawed embryo transfer: a meta-analysis of randomized controlled trials. Laser Med Sci.

[35] Chan DYL, Li TC (2017). Comparison of the external physical damages between laser-assisted and mechanical immobilized human sperm using scanning electronic microscopy. PLoS One.

[36] Liu HC, Xie Y, Gao L, Sun XZ, Liang XY, Deng C, Gao Y, Liu G (2020). Impact on using cryopreservation of testicular or epididymal sperm upon intracytoplasmic sperm injection outcome in men with obstructive azoospermia: a systematic review and meta-analysis. J Assit Reprod Gent.

[37] Eftekhar M, Mohammadi B, Tabibnejad N, Lahijani MM (2020). Frozen-thawed cleavage stage versus blastocyst stage embryo transfer in high responder patients. Zygote..

[38] Li W, Xue X, Zhao WQ, Ren AQ, Zhuo WW, Shi JZ (2017). Blastocyst transfer is not associated with increased unfavorable obstetric and perinatal outcomes compared with cleavage-stage embryo transfer. Gynecol Endocrinol.

[39] Martins WP, Nastri CO, Rienzi L, Poel SZ, Gracia C, Racowsky C (2017). Blastocyst vs cleavage-stage embryo transfer: systematic review and meta-analysis of reproductive outcomes. Ultrasound Obstet Gynecol.

[40] Wang SS, Chen L, Fang JS, Jiang WH, Zhang NY (2019). Comparison of the pregnancy and obstetric outcomes between single cleavage-stage embryo transfer and single blastocyst transfer by time-lapse selection of embryos. Gynecol Endocrinol.

